# Embryonal and non‐meningothelial mesenchymal tumors of the central nervous system – Advances in diagnosis and prognostication

**DOI:** 10.1111/bpa.13059

**Published:** 2022-03-09

**Authors:** David M. Meredith, Sanda Alexandrescu

**Affiliations:** ^1^ Department of Pathology Brigham and Women’s Hospital Harvard Medical School Boston Massachusetts USA; ^2^ Department of Pathology Boston Children’s Hospital Harvard Medical School Boston Massachusetts USA

**Keywords:** CNS embryonal tumors, CNS mesenchymal tumors, methylation, next generation sequencing, WHO classification of CNS tumors

## Abstract

The 5th edition of the WHO Classification of Tumours of the Central Nervous System introduces new entities, and provides updated guidance regarding the diagnostic criteria for tumors of the central nervous system (CNS). CNS embryonal tumors and CNS non‐meningothelial mesenchymal tumors can be challenging for practicing pathologists, as the histologic features are not always specific to a particular entity, and integration of microscopic and molecular findings is necessary. This review on CNS embryonal and non‐meningothelial mesenchymal tumors is meant to provide an update with a focus on WHO changes and additions and on recent discoveries with diagnostic, prognostic, and therapeutic implications.

## Graphical abstract

A concise review of the CNS embryonal and mesenchymal non‐meningothelial tumors, comprising the most current available information with clinical implications.

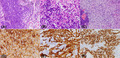

## 1 INTRODUCTION

The goal of this review is to provide a summary of the central nervous system (CNS) embryonal tumors and non‐meningothelial mesenchymal tumors with an emphasis on new discoveries with diagnostic and prognostic implications. CNS embryonal tumors and non‐meningothelial mesenchymal tumors constitute less than 1% of all CNS neoplasms. Their histology and immunophenotype are often non‐specific, which makes them sometimes difficult to diagnose in practice. The development of sophisticated molecular techniques and their implementation in clinical practice led to a precise classification of these tumors and incorporation of many molecularly defined entities in the WHO classification of CNS tumors.

## 2 EMBRYONAL TUMORS

The 5th edition of the WHO Classification of Central Nervous Tumours [1] groups embryonal tumors into medulloblastoma, atypical teratoid/rhabdoid tumor, cribriform neuroepithelial tumor, embryonal tumor with multilayered rosettes, CNS neuroblastoma *FOXR2*‐activated, CNS tumor with *BCOR* internal tandem duplication, and CNS embryonal tumor NEC/NOS. When compared with the 2016 WHO Classification of the Central Nervous Tumours, the 5th edition introduces changes that reflect the most up‐to‐date understanding of CNS embryonal tumors. While the medulloblastoma remains classified based on histologic and molecular subgroups, the 5th edition separates medulloblastomas with SHH activation in different groups based on their *TP53* status; atypical teratoid/rhabdoid tumor (AT/RT) is presented similarly in the 4th and 5th WHO classification, albeit the 5th edition introduces its stratification in AT/RT‐SHH, AT/RT‐MYC, and AT/RT‐TYR, based on DNA methylation and transcriptome signatures; the chapter on embryonal tumors with multilayered rosettes expanded to include rare tumors with *DICER1* mutations; two new entities and a provisional entity were introduced in the 5th edition: CNS neuroblastoma, *FOXR2*‐activated, CNS tumor with *BCOR* internal tandem duplication, and cribriform neuroepithelial tumor, respectively; last but not least, a few entities that existed in the 4th edition of the WHO Classification Of The Central Nervous System Tumours are now encompassed in the entities listed above: CNS neuroblastoma, CNS ganglioneuroblastoma, CNS embryonal tumor NOS. Except for medulloblastomas, which occur in the cerebellum, CNS embryonal tumors can occur anywhere in the neuroaxis. On magnetic resonance imaging (MRI) studies they are heterogeneous mass‐forming tumors that may contain necrotic foci or cysts, and that usually have diffusion restriction.

### 2.1 Medulloblastoma

Medulloblastomas are posterior fossa embryonal tumors that represent 20% of all childhood CNS tumors [2]. They are subclassified into 4 molecular and 4 histologic groups. Molecular groups of medulloblastoma are WNT‐activated, SHH‐activated (*TP53*‐wildtype and *TP53*‐mutant), and non‐WNT/non‐SHH, the latter comprising groups 3 and 4 [1, 3, 4]. Histologically, medulloblastoma exhibits 4 characteristic morphologic patterns: classic, desmoplastic nodular, medulloblastoma with extensive nodularity, and large cell/anaplastic. Although tumors with desmoplastic nodular morphology are highly correlated with SHH activation (described below), the other morphologic patterns are not tightly associated with molecular subclass.

Below is a brief summary of practical points and their implications for the medical care of patients with medulloblastomas:

WNT‐activated medulloblastomas are tumors that arise from the rhombic lip and dorsal brainstem in older children and young adults [5]. They usually have exon 3 activating mutations in *CTNNB1*, or, rarely, *APC* mutations, in which case evaluation for Turcot syndrome is indicated [4, 6]. Monosomy 6 is present in approximately 85% of WNT‐activated medulloblastomas. In practice, pathologists may interrogate for WNT pathway activation via immunohistochemistry for beta‐catenin, which, in WNT‐activated medulloblastomas, is translocated to the tumor nuclei. Of note, nuclear beta‐catenin staining may be present only in a small subset of cells in tumors harboring *CTNNB1* mutations, and thorough evaluation is required for accurate reporting.

WNT‐activated medulloblastomas with classic morphology have very good prognosis with standard therapy, with a 5‐year survival rate of more than 90%. Rarely, large‐cell anaplastic morphology is noted, and the prognostic significance of this finding is not fully known [6]. More recently, rare medulloblastomas harboring dual activation of both SHH and WNT pathways have been described. While these tumors appear to cluster with WNT‐activated medulloblastoma by methylation profiling, it is unclear if such tumors demonstrate similar clinical behavior as conventional WNT‐activated tumors [7].

SHH‐activated medulloblastomas are thought to arise from the cerebellar granule cells [8], and they are further subgrouped on the basis of *TP53* functional status. SHH medulloblastomas have the largest variety of morphologies, with desmoplastic nodular and extensive nodularity highly associated with this molecular group. SHH‐activated medulloblastomas that have intact *TP53* occur mostly in infants and young adults and have good prognosis. Inactivating mutations in the tumor suppressor *PTCH1* is the most common mechanism of SHH upregulation in childhood tumors, whereas mutations in *SUFO* or *SMO* are more common in adults. [4, 6].

SHH‐activated medulloblastoma with *TP53* inactivation occurs in older children and young adults, does not respond to therapy, and has a much worse prognosis. These tumors usually show gene amplification events, such as in *GLI3* or *MYCN* instead of the above listed mutations.

Group 3 medulloblastomas are aggressive tumors that occurs mostly in young children and have a preponderance to present with leptomeningeal dissemination. A subset of group 3 medulloblastomas have amplification of *MYC*, which portends even worse prognosis.

Group 4 medulloblastoma is the most common molecular group, it occurs in older children and adolescents, and is 3 times more common in boys [1]. The prognosis of group 4 medulloblastoma is favorable with standard therapy and uncertain when the histology is large cell/anaplastic [9].

**FIGURE 1:**
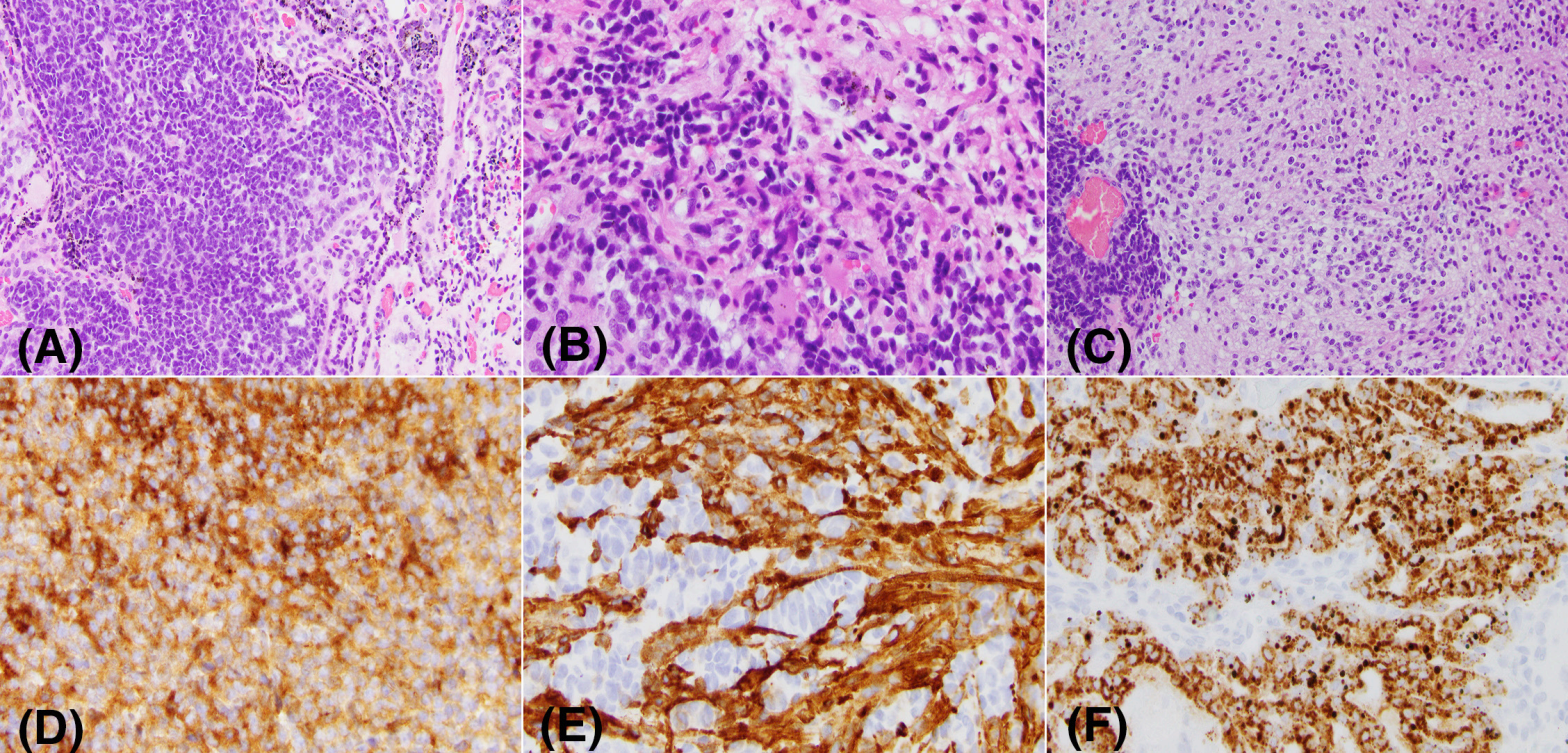
A 2‐year‐old patient with an unusual embryonal cerebellar vermis tumor with myogenic and melanocytic differentiation, with nonspecific copy number changes and no single nucleotide variants of clinical interest; the tumor was placed in the medulloblastoma group 3 category by methylation (A) Embryonal tumor composed of small blue cells with nuclear overlap and atypia, admixed with cord‐like areas containing pigment. (B) Small blue cells, some with abundant eosinophilic cytoplasm and eccentric nuclei (rhabdomyoblastic features). (C) Neuroblastic and more mature, neuronal features with abundant neuropil in the background. (D) Diffuse synaptophysin expression. (E) Desmin expression highlighting skeletal striations. (F) HMB45 expression in a subset of the tumor cells

The modalities for molecular stratification of medulloblastoma vary from institution to institution, with a combination of immunohistochemical stains (beta‐catenin, GLI2, YAP1) and FISH (*MYC* amplification) being most utilized. Nonetheless, an increasing number of institutions are adopting more comprehensive platforms, such as large next generation sequencing panels and chromosomal microarrays to capture diagnostic and prognostic mutational and copy number events. Most medulloblastomas can be stratified in the 4 molecular subgroups based on widely available genomic technologies; however, rare cases have indistinct genetic profiles and are difficult to classify further. In such instances, methylation clustering analysis is a useful method to resolve molecular subgroup status. One such example with myogenic and melanocytic differentiation, which had non‐specific copy number changes and single nucleotide variants of interest is illustrated in Figure 1. Of note, this case had extensive CRX expression, a pineal origin immunomarker, leading to a presumed diagnosis of pineal anlage tumor. This case clustered with medulloblastoma group 3 with high confidence score on methylation analysis.

Although not widely available and not widely clinically validated yet, methylation analysis is a reliable method of distinguishing among the 4 molecular groups of medulloblastoma. One recent study showed the existence of 8 distinct subgroups with prognostic significance in the non‐WNT/non‐SHH group, illustrating the modality's unique ability to resolve otherwise indistinguishable or subtle genomic differences [1, 10].

Histologically, medulloblastoma is subclassified in classic, demosplastic nodular, medulloblastoma with extensive nodularity and large cell/anaplastic variants. Of these, only the large cell/anaplastic variant is an independent indicator of poor prognosis. It can be seen in all molecular groups, but it is mostly encountered in group 3 tumors with *MYC* amplification and should be explicitly reported when present [11].

### 2.2 Atypical teratoid/rhabdoid tumor (AT/RT)

**FIGURE 2:**
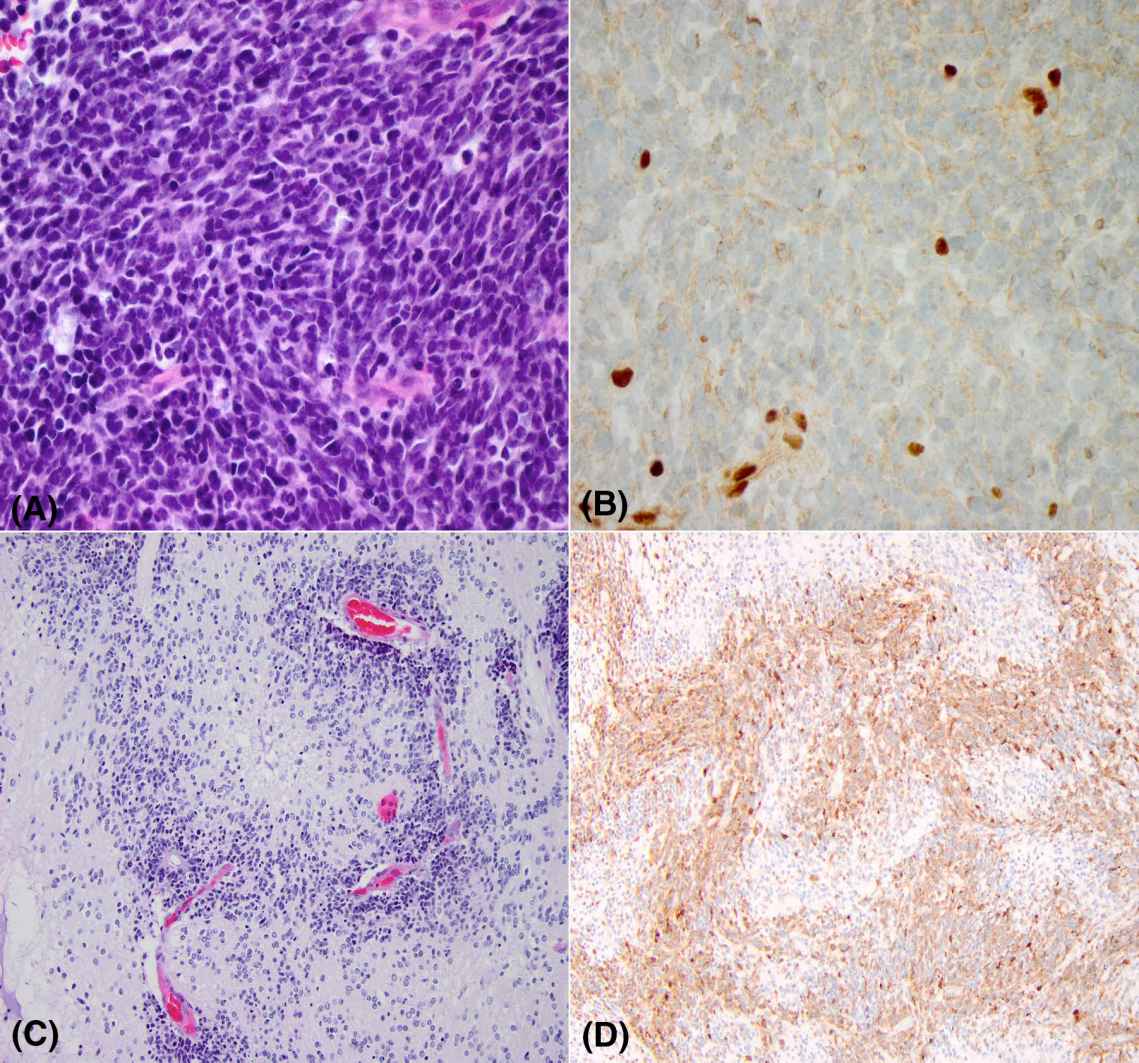
(A) AT/RT composed of small blue cells with overlap and occasional cells with eccentric nuclei; definite classic rhabdoid features were not seen. (B) Loss of INI1 in tumor cells with internal positive control in entrapped cells and vessels. (C) Embryonal tumor with abundant neuropil and true rosettes (ETANTR) with true rosettes surrounded by multiple layeres of neuroblasts, perivascular pseudorosetts, and more cellular areas admixed with less cellular areas that have abundant neuropil. (D) LIN28 diffuse immunoexpression

AT/RT is a rare high‐grade tumor encountered mostly in children younger than 2 years old. It can arise anywhere in the neuroaxis and approximately 25% of cases have leptomeningeal spread at presentation [2, 12]. Although the histology can be quite variable, usually AT/RT contains at least focal regions of rhabdoid cells with eccentric nuclei with prominent nucleoli and with eosinophilic cytoplasm. Mitoses are abundant and necrosis and apoptotic figures are common. They demonstrate patchy expression of GFAP, SMA, EMA and synaptophysin; loss of INI1 or, rarely, BRG1 is required for diagnosis (Figure 2A,B). At a molecular level AT/RT is usually characterized by homozygous deletion of *SMARCB1* (encoding for INI1 protein), or, less frequently, it has a combination of loss‐of‐function mutations and heterozygous deletion of *SMARCB1*. Rarely, there is biallelic alteration of *SMARCA4* (encoding for BRG1 protein).

Recent comprehensive molecular profiling of AT/RT has identified three distinct transcriptional/epigenetic subgroups: AT/RT‐TYR, which is found in the infratentorium in infants, expresses tyrosinase and other melanosomal markers, and may have improved survival; AT/RT‐MYC, which is mostly supratentorial and is seen in older children; and AT/RT‐SHH, which is seen in all locations and demonstrates mutations in SHH and NOTCH pathway. Except for a few long‐term survivors, AT/RT is a devastating disease with a poor prognosis; however, additional investigation is needed to determine if routine molecular subtyping is beneficial for treatment planning and prognosis.

### 2.3 Embryonal tumor with multilayered rosettes (ETMR)

ETMR is a high‐grade predominantly supratentorial primitive tumor that usually occurs in children under the age of 2 years [1]. It is characterized by a chromosome 19 microRNA cluster (C19MC) amplification or fusion with *TTYH1* gene. Histologically, it can have 3 patterns:

- Embryonal tumor with abundant neuropil and true rosettes (ETANTR), which is composed of primitive small blue cells that grow in sheets and multilayered true rosettes admixed with areas of neuropil.
- Ependymoblastoma, which is composed of multilayered rosettes and pseudorosettes with embryonal cells, some with short fibrillary processes; this tumor lacks neuropil.
- Medulloepithelioma, which is composed of embryonal cells with papillary, trabecular or tubular pattern of growth. Some display mesenchymal or melanotic differentiation.

In addition to C19MC alterations, ETMRs are enriched in broad chromosomal alterations, including gains of chromosome 2, as well as 7q, 11 q gains, and 6q loss. Although immunohistochemistry for LIN28A is a reliable, if somewhat nonspecific marker for ETMR (Figure 2C,D), genetic confirmation is advised and may require chromosomal microarray or FISH, as most widely available next generation sequencing panels do not adequately cover the C19MC locus.

More recently, Uro‐Coste et al. [13] described a short series of two cases that histologically resembled ETMR but contained heterologous elements (skeletal muscle and cartilage). Both tumors occurred in the cerebellar vermis and lacked C19MC alterations, but sequencing showed biallelic mutations in the *DICER1* gene, a mechanism indistinguishable to that seen in DICER1 syndrome‐associated tumors. Another study by Lambo et al. demonstrated that *DICER1* alterations in ETMR do not co‐occur with C19MC alterations. Furthermore, it highlighted that rare ETMRs without C19MC or *DICER1* alterations, have amplification of the miR‐17‐miRNA cluster on chromosome 13, or clustered breakpoints that affected the C19MC locus suggesting that, even if C19MC was not amplified, occult rearrangement events may still lead to upregulation. [14].

Irrespective of morphology, the prognosis of ETMR is poor, with most patients dying of their disease within a year [15, 16].

### 2.4 CNS neuroblastoma, FOXR2‐activated

**FIGURE 3:**
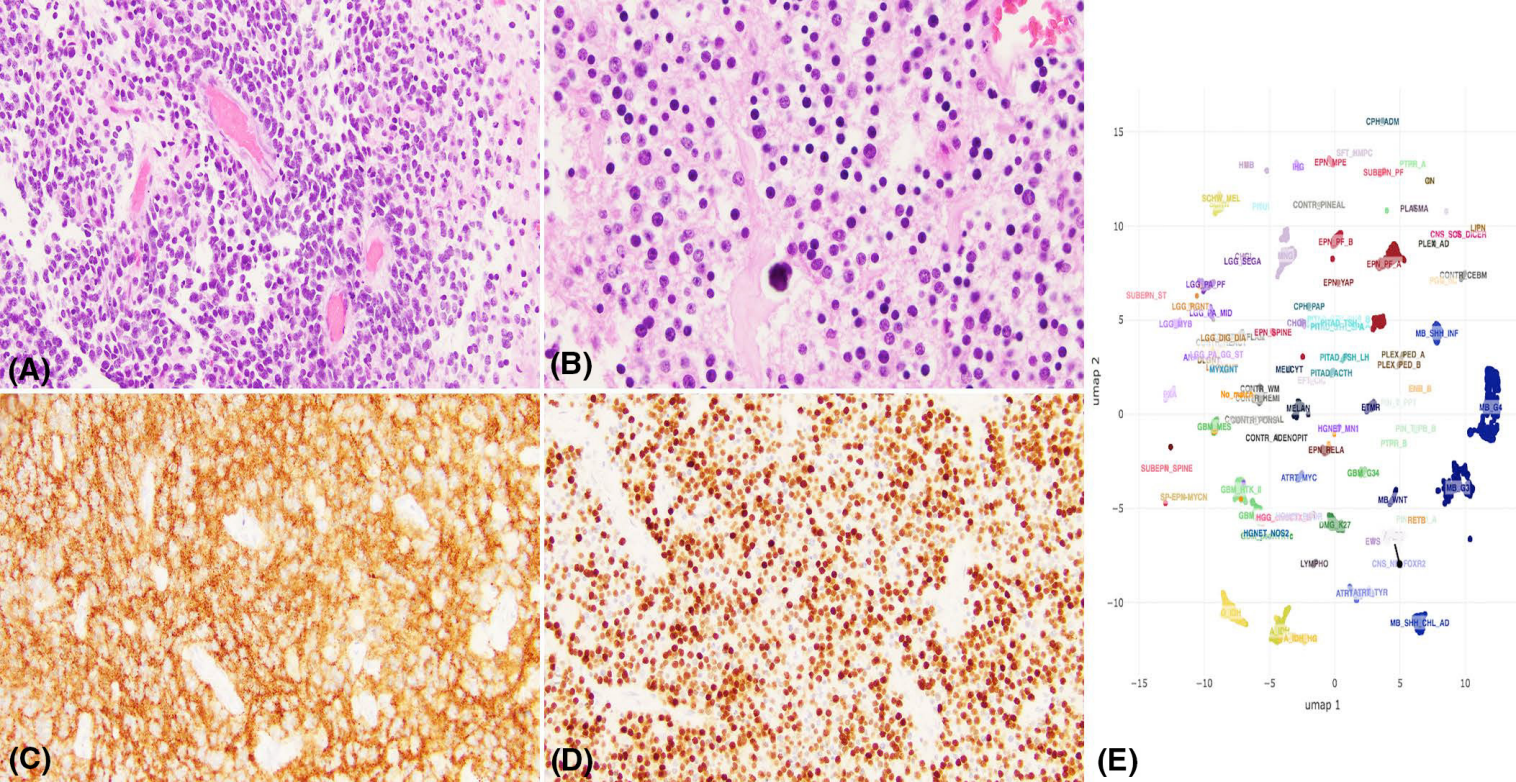
CNS neuroblastoma with FOXR2 alteration. (A) Primitive small blue cells with nuclear overlap and karyomegaly in a background of neuropil. (B) oligo‐like areas with calcification. (C) Diffuse synaptophysin expression. (D) Diffuse OLIG2 expression. (E) Diagram showing this case being placed with the reference methylation group of CNS neuroblastoma FOXR2‐altered. This case is courtesy to Dr. Hart Lidov, Department of Pathology, Boston Children's Hospital, and Dr. Kenneth Aldape, National Institute of Health

CNS neuroblastoma, *FOXR2*‐activated was described by Sturm D. et al. [17] when it was observed that a subset of the tumors diagnosed in the past as CNS primitive neuroectodermal tumors have *FOXR2* rearrangements and overlapping methylation profiles. These tumors arise in the cerebral hemispheres or along the ventricular system, have small cell morphology in a background of neuropil, and express mixed markers such as OLIG2 and synaptophysin. Many contain neurocytic or ganglion cells, as well as Homer Wright rosettes and perivascular pseudorosettes. In addition to *FOXR2* alterations, 1q gain and 16q loss are common. The *FOXR2* alterations encountered are usually complex and not easily identified even with existing next generation sequencing methods; hence methylation analysis may be helpful in such cases. An example of CNS neuroblastoma with *FOXR2* alteration is illustrated in Figure 3.

### 2.5 CNS tumor with *BCOR* internal tandem duplication

**FIGURE 4:**
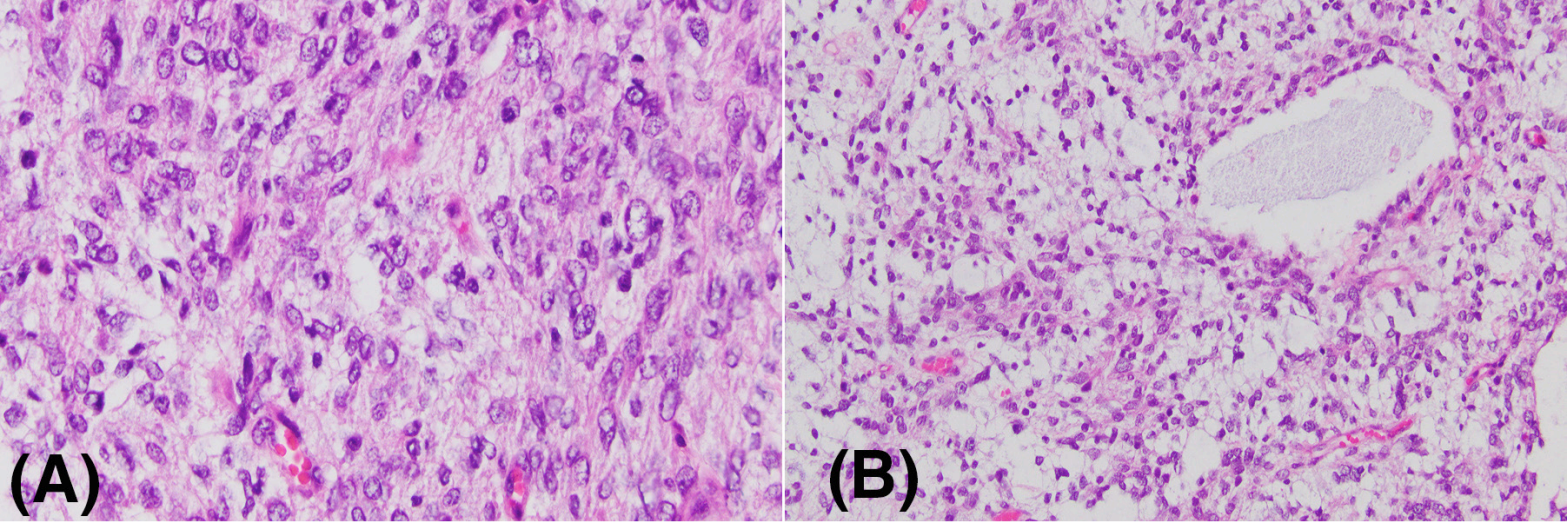
CNS neuroepithelial tumor with *BCOR* ITD: (A) Hypercellular sheets of markedly atypical cells with open chromatin and karyomegaly and mitoses. The cytological features are relatively nonspecific. (B) Less cellular areas with abundant myxoid background stroma and microcysts

Like the above entity, another subset of CNS PNET with distinct methylation profile was shown to harbor recurrent internal tandem duplications (ITD) within the terminal exon of *BCOR*. These CNS tumors with *BCOR* (ITD) are malignant, predominantly solid tumors composed of oval or spindle cells that grow in sheets and ependymal‐like perivascular pseudorosettes in a background that sometimes can be myxoid and microcystic (Figure 4) [18, 19]. They usually occur in the cerebral and cerebellar hemispheres and appear well demarcated, albeit focal infiltration of surrounding brain parenchyma can be present. The immunophenotype is non‐specific, as it may show patchy expression of OLIG2, GFAP, S100, NeuN, and synaptophysin, but not to the extent seen in most gliomas or embryonal tumors. Diffuse strong nuclear expression of BCOR by immunohistochemistry is present, but molecular confirmation is needed, as patchy immunopositivity may be encountered in other tumors irrespective of *BCOR* mutational status, including solitary fibrous tumors [20]. Although the data on outcomes is limited, most patients with CNS tumors with *BCOR* ITD have relapses and poor prognosis.

### 2.6 Provisional entity: Cribriform neuroepithelial tumor (CRINET)

The 5th Edition of the WHO Classification of CNS Tumours [1] introduced CRINET as a provisional diagnosis; it is a non‐rhabdoid neuroectodermal tumor with a prominent cribriform arrangement of tumor cells that have characteristic loss of INI1 due to homozygous loss‐of‐function of *SMARCB1*. Although CRINET is placed by methylation analysis in the same group as AT/RT‐TYR, the prognosis seems to be better in the few cases that have been described [21, 22] and has been therefore provisionally separated as an entity from AT/RT.

As defined by the cIMPACT‐NOW and by the WHO, tumors without molecular characterization should be named “not otherwise specified (NOS), and tumors for which extensive molecular testing was performed but no driver was identified, should be named “not elsewhere classified (NEC)”.

## 3 CNS NON‐MENINGOTHELIAL MESENCHYMAL TUMORS

Aside from meningioma, primary mesenchymal tumors of the CNS are rare and comprise a diverse group of entities. Those included in the 5th Edition of the WHO Classification of CNS Tumours represent mostly tumors for which the overlap with the soft tissue counterpart is minimal. In the WHO blue book, mesenchymal non‐meningothelial tumors of the CNS are grouped into:

- Fibroblastic and myofibroblastic tumors: solitary fibrous tumor.
- Vascular tumors: hemangiomas, vascular malformations, and hemangioblastoma.
- Skeletal muscle tumors: rhabdomyosarcoma.
- Tumors of uncertain differentiation: intracranial mesenchymal tumor, FET:CREB fused, CIC‐rearranged sarcoma, primary intracranial sarcoma, DICER1‐mutant, and Ewing sarcoma.
- Chondro‐osseous tumor; mesenchymal chondrosarcoma, chondrosarcoma.
- Notochordal tumors: chordoma.

The list of mesenchymal non‐meningothelial tumors included in the 5th edition of the WHO is abbreviated compared to the list in the 4th edition, as entities that are common in soft tissue pathology are presented in the WHO Classification of Bone and Soft Tissue Tumours. The entity hemangiopericytoma/solitary fibrous tumor is now simply named solitary fibrous tumor. In addition, a few tumors described recently in the literature are recognized as new entities: intracranial mesenchymal tumor *FET‐CREB* fusion‐positive, *CIC*‐rearranged sarcoma, and primary intracranial sarcoma, *DICER1*‐mutant.

### 3.1 Fibroblastic and myofibroblastic tumors

The histology and molecular features of solitary fibrous tumor are well known and will be only briefly summarized here. Solitary fibrous tumor is a dura‐based fibroblastic neoplasm that represents less than 1% of all CNS neoplasms. It is characterized by *NAB2‐STAT6* gene fusion and STAT6 immunoexpression [23, 24]. Histologically, it is composed of spindled and ovoid monomorphic cells arranged in fascicles and sheets admixed with hyalinized, dilated, branching blood vessels. The cellularity can be variable, with some tumors having less cells and abundant collagenized stroma, while others are highly cellular and have a patternless architecture. In addition to *NAB2‐STAT6* fusion, *TERT* promoter mutations and *TP53* mutations have been identified in a subset of solitary fibrous tumors with more aggressive biology [25]. The CNS solitary fibrous tumors tend to recur and metastasize even many years after the initial diagnosis; the only histologic features that are predictive of worse prognosis are the mitotic rate and presence of necrosis.

In addition to CNS solitary fibrous tumor, which is included in the WHO classification of CNS tumors, there are other fibroblastic/myofibroblastic tumors that are not included in the WHO book, but that occur in practice, albeit less frequently. A review of these tumors is provided below.

### 3.2 Inflammatory myofibroblastic tumor (IMT) and other fibroblastic/myofibroblastic neoplasms

IMT is usually encountered in the lung, retroperitoneum or pelvis, but a limited number of cases has been described in the CNS [26, 27, 28, 29]. IMT is a rare tumor of intermediate biologic potential that, in the brain, tends to involve the dura and leptomeninges. It is composed of a population of atypical myofibroblasts in a background of mixed inflammation [30]. ALK immunoexpression and *ALK* fusions in IMT‐CNS have been estimated at approximately 30%.[26]. IMT tends to recur locally and to increase morbidity overtime.

**FIGURE 5:**
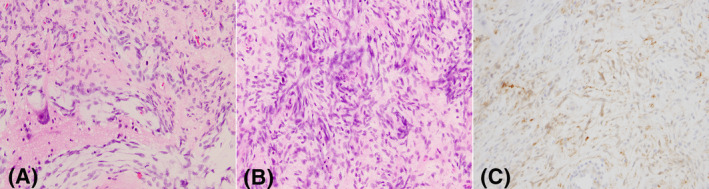
CNS fibroblastic tumor with *NTRK* rearrangement. (A) Spindle cell neoplasm invading the brain tissue. (B) Hypercellular clusters of spindle cells with a paternless pattern. Mitoses were difficult to find. (C) pan‐TRK antibody showing pale but diffuse positivity in the tumor cells

*NTRK* alterations have been described in a variety of tumor types, including pediatric fibroblastic/myofibroblastic tumors [31], high and low‐grade gliomas [32], papillary thyroid carcinoma, lung adenocarcinoma, and secretory breast adenocarcinoma [33]. Torre et al. [34] described a case of a fibroblastic tumor with *NTRK* rearrangement primary in the CNS; it occurred in a 20‐month‐old child, involved the cortex and leptomeninges of the left temporal lobe, and had a meningioangiomatosis pattern of growth along the Virchow Robin spaces. (Figure 5). But unlike meningioangiomatosis, immunoreactivity for a pan‐TRK antibody was diffusely positive, in keeping with the fusion status. The morphology and molecular alteration of this tumor was reminiscent of those seen in extracranial fibroblastic tumors with *NTRK* rearrangements, which are tumors of intermediate grade, with a propensity to recur if not completely resected. At the last follow‐up, 2 years after the presentation, the patient was free of disease, but it is unclear how primary CNS fibroblastic tumors with *NTRK* rearrangements behave long term.

### 3.3 Vascular tumors, comprising hemangiomas, vascular malformations and hemangioblastomas

#### 3.3.1 Hemangiomas and vascular malformations

**FIGURE 6:**
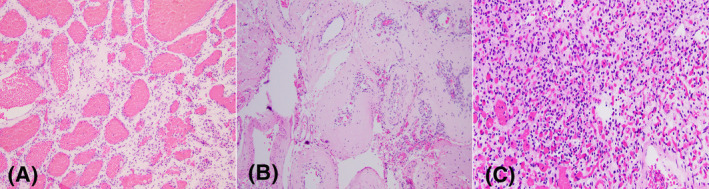
(A) Hemangioma‐like vascular anomaly in the setting of PIKC3A overgrowth syndrome: back‐to‐back dilated vascular lumens with delicate tortuous walls. Case courtesy to Dr. Hart Lidov, Department of Pathology, Boston Chidldren's Hospital. (B) A case of arteriovenous malformation with *BRAF V600E* mutation: abnormal vessels with walls with venous and arterial quality. This vascular anomaly involved the leptomeninges and dissected into the grey matter, too. (C) Hemangioblastoma, composed of sheets of clear cells admixed with abundant delicate vascular channels

Cerebral hemangiomas are benign vascular neoplasms frequently composed of multiple back‐to‐back capillary‐type vessels. They can be isolated or be part of a *PIK3CA*‐related overgrowth syndrome. Filippidis A. et al. described recently a pediatric patient with macrocephaly and facial dysmorphic features who was found to be mosaic for *PIK3CA R108H*. A brain MRI was performed part of routine screening for overgrowth syndrome, and it uncovered a large parafalcine mass that radiographically mimicked meningioma. Histologic examination showed back‐to‐back dilated vessels with thin walls and occasional calcifications, in keeping with a vascular lesion, and there were no meningothelial cells seen (Figure 6A) [35].

Cavernous malformations are solitary or, rarely, multifocal vascular anomalies composed of back‐to‐back sinusoidal vessels with fibrotic walls and intraluminal thrombi. There are no arterial or venous features, and they have very little to no intermixed brain tissue. Familiar forms are associated with mutations in *KRIT*, *CCM2* and *PDCD10* [36].

Cerebral arteriovenous malformations are sporadic fast‐flow vascular anomalies composed of malformed arteries and veins with intervening brain parenchymal with gliosis and hemosiderophages. Recent studies demonstrated somatic *KRAS* and *BRAF* mutations in a subset of these lesions [37]. A case of an arteriovenous malformation with a *BRAF V600E* mutation is illustrated in Figure 6B.

Hemangiomas usually do not recur after complete resection. Cavernous malformations have an annual risk of hemorrhage of 1.6–4.6%, and previous hemorrhage and brainstem location are associated with increased risk of bleeding [38]. In AVM, the risk of hemorrhage is of 2%–5% per year, and, if hemorrhage happens, the risk of death is up to 25% [39].

#### 3.3.2 Hemangioblastoma

Hemangioblastoma is a benign vascular tumor composed of neoplastic stromal cells with clear cytoplasm, characteristic Inhibin positivity, and *VHL* gene alterations (Figure 6C). It is assigned a WHO grade 1. Hemangioblastomas are rare, accounting for less than 2% of all brain tumors. They may occur sporadically or in the setting of von Hippel‐Lindau syndrome [40]. The most common location is the cerebellum, but they can be encountered rarely in the spinal cord, retina, peripheral nerves or even outside the CNS [41]. The prognosis of hemangioblastoma is excellent, particularly in completely resected sporadic cases.

### 3.4 Tumors of uncertain differentiation

#### 3.4.1 Intracranial mesenchymal tumor, FET‐CREB fusion‐positive

**FIGURE 7:**
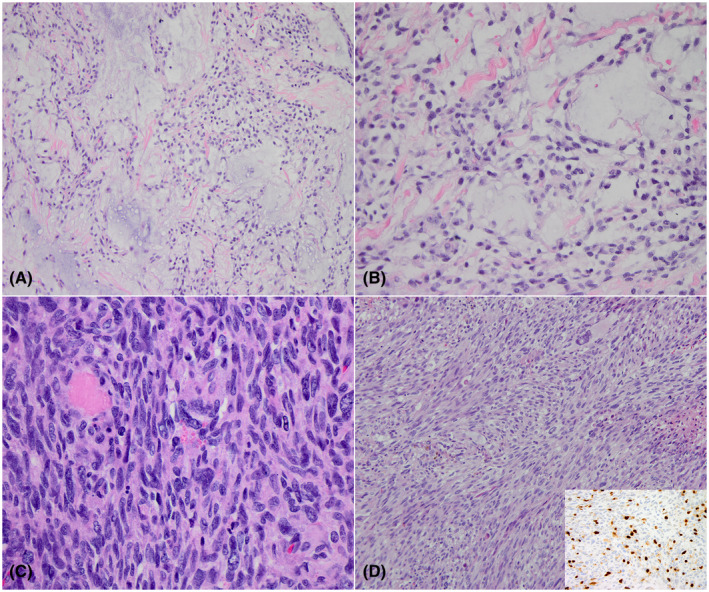
(A and B) An intracranial mesenchymal tumor with *EWSR1‐CREB1* fusion, composed of round‐to‐oval cells with vacuolar cytoplasm and mild nuclear atypia in a myxoid background stroma admixed with collagen fibers. (C and D) Primary CNS sarcoma, *DICER1*‐mutant composed of pleomorphic cells, some with eosinophic granules (C). The cells are arranged in sheets (C) and fascicles (D)

Intracranial mesenchymal tumor, FET‐CREB fusion‐positive was introduced in the 5th Edition of the WHO classification of CNS tumors as a provisional entity. It is a mesenchymal neoplasm characterized by fusion of a FET family member, usually *EWSR1*, with a member of the CREB family of transcription factors (*CREB1*, *ATF1* or *CREM*). Usually they are supratentorial, well delineated, extra‐axial tumors that occur in children and young adults [42, 43]. The morphology features of these CNS tumors show modest correlation with the gene fusion partner; nonetheless, most cases exhibit a collagenous stroma and epithelioid to stellate or spindle cells. Collections of thin‐walled vessels and peripheric lymphoplasmacytic infiltrate and hemosiderin deposits are also common findings [44]. The immunohistochemical profile is variable, but usually includes some degree of positivity for EMA, CD99 and desmin. A case is illustrated in Figure 7A,B. The variable morphology and nonspecific molecular profile make this tumor difficult to diagnose in the absence of molecular testing and demonstration of a *FET‐CREB* gene fusion. It is uncertain how this tumor is linked to soft tissue angiomatous fibrous histiocytoma, primary pulmonary myxoid sarcoma, hyalinizing clear cell sarcoma of the salivary gland and gastrointestinal clear cell sarcoma, all of which demonstrate similar fusions. The prognosis is uncertain, as the number of cases reported is low; some of the reported cases had an indolent course, while others recurred rapidly [44, 45].

#### 3.4.2 Primary intracranial sarcoma, DICER1‐mutant

Primary intracranial sarcoma, DICER1‐mutant, is a new entity in the 5th edition of the WHO. Koelsche et al. published an extensive study describing a group of CNS sarcomas previously classified as gliosarcoma, glioblastoma, malignant tumor, extra‐skeletal mesenchymal chondrosarcoma and primitive neuroectoderma tumor, which all displayed similar methylation patterns and possessed *DICER1* inactivating mutations. Histologically, these tumors were composed of spindle and pleomorphic cells arranged in fascicles admixed with areas of reduced cellularity. All had immunohistological evidence of rhabdomyoblastic differentiation, and some contained chondroblastic elements. All these tumors had high mitotic rate and areas of necrosis [46]. Later on, Lee et al. [47] described a series of primary intracranial sarcomas with *DICER1* mutations, showing that the most common location is in the cerebral hemispheres, that histologically they invariably contain intracytoplasmic eosinophilic granules, and that, in addition to the *DICER1* mutations, *TP53* mutations and Ras pathway activation were common, and can occur in the setting of Neurofibromatosis type 1. Alexandrescu et al. [48] highlighted the variable histology of these tumors and demonstrated that they have loss of H3K27me3 as well as TLE1 expression. Because the histology of these tumors is highly variable and the differential diagnosis vast, confirmation of the *DICER1* mutations via sequencing is invariably needed for a firm diagnosis. The prognosis for patients with *DICER1*‐mutant primary intracranial sarcoma remains unknown given the hitherto small series. An aggressive course is suspected, but there is no long‐term follow‐up data reported yet.

In addition to the new WHO entities mentioned above, there are bone and soft tissue sarcomas that are described outside of the central nervous system and that can also occur in the brain: rhabdomyosarcoma, *CIC*‐rearranged sarcoma, Ewing sarcoma, mesenchymal chondrosarcoma, chodrosarcoma, chordoma, etc. Among these, *CIC*‐rearranged sarcoma was relatively recently described in the brain, and hence will be briefly summarized below.

#### 3.4.3 CIC‐rearranged sarcoma

CIC‐rearranged sarcoma is a high‐grade mesenchymal tumor that usually occurs in the viscera, but may rarely occur within the CNS as well. All *CIC*‐rearranged sarcomas have a fusion of *CIC* transcriptional repressor with various partners, most often *DUX4*, but also *NUTM1* or *NUTM2* have been observed [36, 38, 49]. While in most peripheral *CIC*‐rearranged sarcomas the fusion partner is *DUX4*, in the brain the most common fusion partner is *NUTM1* [50, 51]. Histologically, it is a small round blue cell tumor with increased mitotic rate with a background desmoplastic stroma with occasional myxoid areas. The tumor cells express ETV4, WT1 and CD99, while NKX2‐2 is typically negative, which helps in the distinction from Ewing sarcoma. The outcomes of CNS *CIC*‐rearranged sarcoma are not well published; the tumors in the soft tissues have an aggressive course and poor response to therapy.

Despite the rarity of CNS embryonal and non‐meningothelial mesenchymal neoplasms, large scale molecular profiling efforts continue to identify novel entities with distinct clinicopathologic features, expanding the already wide array of tumors that neuropathologists encounter. This review discusses the most recent updates with diagnostic, prognostic, and therapeutic implications as they pertain to embryonal and non‐meningothelial mesenchymal tumors of the brain to improve their recognition and selection of appropriate ancillary testing for this diverse group of entities.

## CONFLICTS OF INTEREST

The authors have no conflicts of interest to disclose.

## AUTHOR CONTRIBUTIONS

David M. Meredith contributed a large part of the text and references for the section on non‐meningothelial mesenchymal tumors and edited the manuscript. Sanda Alexandrescu contributed the text and references for the section on embryonal tumors and a part of the non‐meningothelial mesenchymal tumors, constructed the figures, and submitted the manuscript.
